# Why do women prefer home births in Ethiopia?

**DOI:** 10.1186/1471-2393-13-5

**Published:** 2013-01-16

**Authors:** Solomon Shiferaw, Mark Spigt, Merijn Godefrooij, Yilma Melkamu, Michael Tekie

**Affiliations:** 1 School of Public Health, Addis Ababa University, PO Box 9086, code 1000, Addis Ababa, Ethiopia; 2 Department of General Practice, CAPHRI School for Public Health and Primary Care, Maastricht University, Maastricht, Netherlands; 3 Department of General Practice, Tromso University, Tromso, Norway; 4 Technical Advisor-GCACP, International Planned Parenthood Federation, Nairobi, Kenya; 5 UNFPA-Ethiopia Country Office, Addis Ababa, Ethiopia

**Keywords:** Home births, Traditional birth attendants, Preference, Place of delivery, Ethiopia

## Abstract

**Background:**

Skilled attendants during labor, delivery, and in the early postpartum period, can prevent up to 75% or more of maternal death. However, in many developing countries, very few mothers make at least one antenatal visit and even less receive delivery care from skilled professionals. The present study reports findings from a region where key challenges related to transportation and availability of obstetric services were addressed by an ongoing project, giving a unique opportunity to understand why women might continue to prefer home delivery even when facility based delivery is available at minimal cost.

**Methods:**

The study took place in Ethiopia using a mixed study design employing a cross sectional household survey among 15–49 year old women combined with in-depth interviews and focus group discussions.

**Results:**

Seventy one percent of mothers received antenatal care from a health professional (doctor, health officer, nurse, or midwife) for their most recent birth in the one year preceding the survey. Overall only 16% of deliveries were assisted by health professionals, while a significant majority (78%) was attended by traditional birth attendants. The most important reasons for not seeking institutional delivery were the belief that it is not necessary (42%) and not customary (36%), followed by high cost (22%) and distance or lack of transportation (8%). The group discussions and interviews identified several reasons for the preference of traditional birth attendants over health facilities. Traditional birth attendants were seen as culturally acceptable and competent health workers. Women reported poor quality of care and previous negative experiences with health facilities. In addition, women’s low awareness on the advantages of skilled attendance at delivery, little role in making decisions (even when they want), and economic constraints during referral contribute to the low level of service utilization.

**Conclusions:**

The study indicated the crucial role of proper health care provider-client communication and providing a more client centered and culturally sensitive care if utilization of existing health facilities is to be maximized. Implications of findings for maternal health programs and further research are discussed.

## Background

With an estimated 342,900 deaths worldwide in the year 2008, maternal mortality – the death of a woman while pregnant or within 42 days of termination of pregnancy – remains one of the most formidable challenges facing the developing world [1]. Ethiopia is among the top six high burden countries in which half of global maternal deaths occur, with an estimated maternal mortality ratio of 470 per 100, 000 live births [1,2]. The major causes of maternal death in low income countries include obstructed labor, ruptured uterus, severe pre-eclampsia/eclampsia, malaria and complications from abortion [3-6].

Skilled attendants during labour, delivery and in the early postpartum period, can prevent up to 75% or more of maternal deaths [7,8]. However, according to the most recent Ethiopian Demographic and Health Survey (EDHS), very few mothers (34%) make at least one antenatal visit and even less receive delivery care from skilled professionals. Twenty-eight percent of births were assisted by a traditional birth attendant (TBA) and 57 percent of births were assisted by a relative, or some other person [2]. The World Health Organization defines TBA as “a person who assists the mother during childbirth and who initially acquired her skills by delivering babies herself or by working with other TBAs”. TBAs are often older women and are generally illiterate [9].

The large body of evidence on factors contributing to poor delivery service utilization comes from quantitative studies, which consistently report physical and financial barriers as well as low social status of women as important barriers [10-13].

Many low income countries aim to decrease maternal mortality through implementing multifaceted interventions, including improved access to emergency obstetric services [14]. However, there are instances where hospitals with trained professional staff exist, and yet maternal mortality remains unacceptably high [15] indicating the mere availability of obstetric services does not necessarily result in better maternal health [16,17].

Although there is recognition that responsiveness of care could be an important determinant of maternal mortality, there is little empirical evidence on the extent and importance of such factors in many low income countries. Responsiveness of care usually includes respect towards the patient as reflected by the degree to which the health system is sensitive to patients’ dignity, confidentiality, and autonomy as well as the level of attention given to clients (promptness, quality of environment, access to social assistance and free choice of provider) [18].

The present study reports findings from Ethiopia, where Key challenges related to transportation and availability of obstetric services were addressed by an ongoing project [19], giving a unique opportunity to understand why women might continue to prefer home delivery even when facility based delivery is available at minimal cost. In this paper we report on the barriers to health facility delivery identified in a setting where finance and distance play lesser roles, with the aim of providing clear directions for future intervention programs in similar circumstances.

## Methods

### Setting

The study took place in a predominantly rural area (Kembatta-Tembaro) in Ethiopia. The area has an estimated total population of 1,266,275 people at the time of the study. There were 11 Health centers (9 public, 2 Non Governmental Organization), 126 Health Posts and 13 private clinics (medium and small including drug stores) within the area. A health center is a midlevel health facility catering for a population of approximately 25,000 while a health post is the smallest publicly owned health facility serving up to 5,000.

Worth to note is the fact that the study was conducted around a health facility which was upgraded to provide comprehensive emergency obstetric care to the surrounding population. Specifically, the renovated health center had an experienced obstetrician-gynecologist and an ambulance that provided 24 h service either free or at minimal charge which was made available through a project support [19]. The Health center has 15 nurses (including one midwife), one public health officer, one laboratory and pharmacy technician, one anesthetist, one pediatrician and a Gynecologist/obstetrician. Most health centers in Ethiopia (including those around the study area) are staffed by health officers, nurses/midwives and laboratory and pharmacy technicians and generally have no specialists in Gynecology/obstetrics or pediatrics.

### Study design and sampling

The study used a mixed study design employing a cross sectional household survey among 15–49 year old women combined with qualitative approaches (in-depth interviews and focus group discussions (FGDs)). The quantitative survey employed a cluster sampling scheme selecting representative samples from each of the three districts using the probability proportional to sample (PPS) technique. Key informants and focus group discussion participants were selected purposively and saturation of information was used to decide on adequacy of the samples. The study got ethical clearance from the respective Health Bureau, and informed verbal consent was obtained from all participants.

### Study participants and survey instruments

A pre-tested structured questionnaire developed in the local language was used to collect information on awareness about and utilization of maternity care services (antenatal, delivery and postnatal) and reasons for not seeking delivery care. The questions were adapted from previous surveys [2].

Overall, a total of three focus group discussions were conducted. The discussions involved three separate groups with women, men and community health workers who opted for institutional or home delivery for the most recent births. As shown in Table 1, a total of 23 people participated in the three focus group discussions. Additionally, in-depth interviews were held with six health care providers (physician, nurses, and a health officer) and two TBAs.

**Table 1:** Characteristics of participants in focus group discussions and in-depth interviews

	**Background characteristics**		
**Participant category**	**Age (Mean, range)**	# **of children (Mean, range)**	# **with recent home delivery**
**Community**			
Mothers - FGD - 8 participants	30 (25, 35)	4.8 (3, 7)	4
Fathers - FGD - 8 participants	36 (28, 40)	6.2 (1, 9)	4
**Community health workers/professionals**	**Type (number)**		**Gender**
Volunteer community health workers	FGD −7 participants		3 females
Traditional Birth Attendants	Interviews - 2 participants		2 females
Midwife/nurse/health officer	Interviews (4 nurses; 1 health officer)		4 females
Gynecologist-Obstetrician	Interview – 1 participant		1 male

The questions included, among others, issues affecting institutional delivery including how and who makes decisions regarding place of delivery, perceived barriers to skilled attendance at delivery, experiences in relation to the behavior and competency of the health care providers, satisfaction with quality of the service, as well as relationships between traditional birth attendants and the formal health system.

The study teams consisted of three graduate students in public health and 30 experienced enumerators who have completed at least 12 years of secondary education. The research teams participated in a two-days training session that included: study overview, ethical conduct of research, role play and pre-testing the study instruments.

### Data analyses

Interviewers took extensive notes, in addition to tape recording and transcribing the interviews. The transcripts were reviewed by the research team. All FGDs and in-depth interviews were translated into English by the research assistants who led the interviews/discussions. Qualitative data were analyzed through thematic analysis using OpenCode software [20,21]. Coding of the transcriptions line by line was done by the Principal Investigator and the second co-author who had previous research experience in maternal health and qualitative data analysis. In many instances, verbatim quotations (sometimes slightly modified from the original translation to make the English more understandable) are used to illustrate responses on relevant issues and themes.

### Results

#### Maternity service utilization

A total of 909 women in the age group 15–49 years participated in the household survey. As Table 2 shows, the majority of respondents was married (75%), less than 30 years of age (54%) with only primary level of education (50%).

**Table 2:** Socio-demographic profile of respondents, Kenbata-Tembaro zone, Southern Ethiopia

**Characteristics**	**Number**	**Per cent**
**Age**		
15-29	488	53.7
30-39	321	35.3
40-49	100	11.0
**Education status**		
No formal education	313	34.4
Primary	450	49.5
Secondary and above	146	16.1
**Marital status**		
Married	680	74.8
Divorced	4	0.4
Widowed	25	2.8
Separated	5	0.6
Never married	195	21.5
**Total**	**909**	**100**

Seventy one percent of mothers received antenatal care from a health professional (doctor, health officer, nurse, or midwife) at least once for their most recent birth in the one year preceding the survey. On average, pregnant women made 3 antenatal care visits. As shown in Table 3, the overwhelming majority of births (84%) in the one year preceding the survey were delivered at home. The proportion of mothers delivering at home did not differ significantly by educational status or household decision making capacity, though women with at least secondary education were more likely to deliver in an institution (p-value > 0.05). Women who reported that they were able to decide about household expenditure by themselves or jointly with their husbands were also slightly more likely to deliver in a health facility, though this was not statistically significant (p-value > 0.05).

**Table 3:** Percentage distribution of births in the last one year preceding the survey by type of professionals and place of delivery, according to background characteristics

**Characteristics**	**Place of delivery**		**P**-**value**	**Health professional**	**Type of Professionals**			**P**-**value**	**Total**
	**Home**	**Institutional**			TBA*	HEWs**	**No one**		
**Age**									
15-29	81.9	18.1	0.58	18.9	76.5	3.0	1.5	0.49	132
30-39	86.8	13.2		13.6	78.6	1.9	5.8		103
40-49	83.3	16.7		16.7	83.3	0.0	0.0		12
**Educational status**									
No formal education	84.1	15.9	0.37	13.2	75	5.9	5.9	0.10	68
Primary	85.5	14.5		15.9	80.8	1.3	2		151
Secondary and above	75.0	25.0		28.6	67.9	0	3.6		28
**Decision on household expenses** (**n**=**239**)									
Respondent	85.7	14.3	0.19	14.3	85.7	0	0	0.79	7
Husband	87.9	12.1		14.4	77.5	3.6	4.5		111
Jointly	79.2	20.8		19.8	76	1.7	2.5		121
**Total**	**84**.**0**	**16**.**0**		**16**.**6**	**77**.**7**	**2**.**4**	**3**.**2**		**247**

* - Traditional Birth Attendant ** - HEWs - Health Extension Workers.

Overall only 16% of deliveries were assisted by health professionals, while a significant majority (78%) was attended by traditional birth attendants. As showed in Table 3, Health Extension Workers (HEWs) attend only a small percentage (2.4%) of deliveries. Health Extension Workers are female community health workers with one year training and work at local health posts providing a package of essential interventions to meet the community health needs at the village level. Two HEWs are assigned in each village (which on average has 1000 households) and are assisted by voluntary community health workers who are expected to serve 50 households.

Births to younger mothers (less than 30) and those with a higher level of education were more likely to be assisted by trained health professionals. Women who reported to make joint decisions on household expenses were more likely to have skilled attendance at delivery compared to women who reported that their husbands (or by themselves) make such decisions (20% versus 14% respectively). However, the associations between education and women’s decision making capacity and delivery by health professionals were not statistically significant (p-value > 0.05).

#### Reasons for preferring home delivery

**Figure 1:**
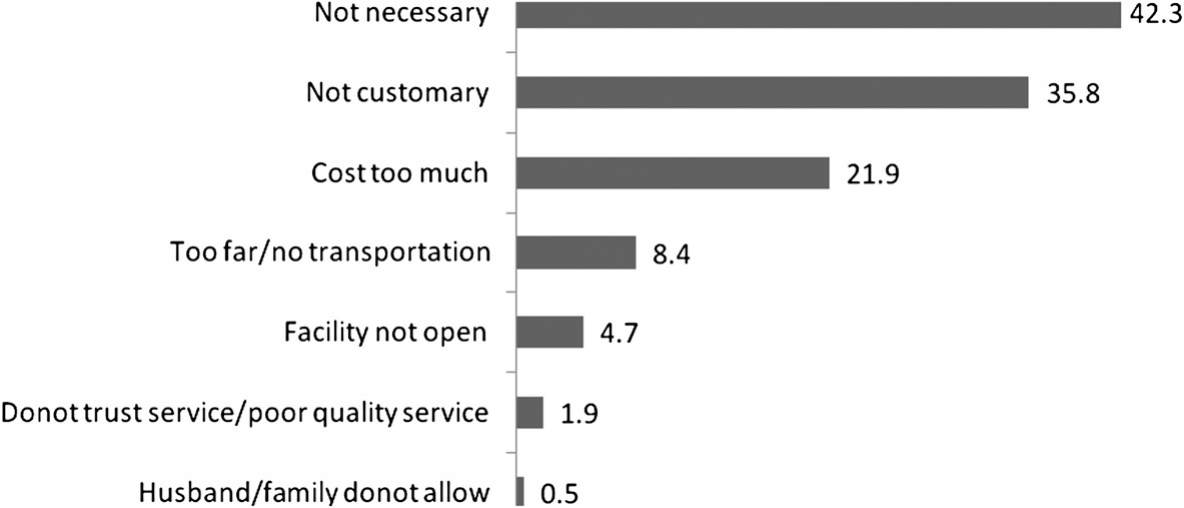
Percentage of women with specific reasons for giving birth outside Health Institutions, (n =215), Kenbata -Tembaro Zone, Southern Ethiopia**.

Women who gave birth outside health institutions were asked whether any of the following factors might be concerns; obtaining money for the service fee, distance to a health facility or lack of transportation, getting permission from husband/family to go for treatment, trust in service quality, belief that it may not be necessary or customary. Figure 1 shows that the most important reasons for not seeking institutional delivery are the belief that it is not necessary (42%) and not customary (36%), followed by high cost (22%) and distance or lack of transportation (8%).

#### Findings from focus group discussions and in-depth interviews

As shown in Table 4, recurring themes that evolved from the group discussions and interviews were related to perception of traditional birth attendants as culturally acceptable and competent health workers, perceived quality of care and previous negative experiences with health facilities, low awareness and less empowered women, as well as economic constraints during referral.

**Table 4:** Themes and sub-themes emanating from FGDs and in depth interviews about barriers to delivery in health facilities

**Main themes**	**Sub themes**
1. TBAs as culturally acceptable and competent health workers	Sensitive to customary practices
	Ability of TBA to handle normal deliveries
	Income and incentives of TBAs
	Support from family members during delivery
2. Perceived quality of care and previous negative experiences	Incompetent health workers
	Out of hours availability
	Health workers do not allow cultural practices
	No attention for privacy and psychosocial support
	Shortage of female health workers
3. Low awareness and less empowered women	Believe skilled attendance is unnecessary
	Decision making by spouses, relatives, and neighbors
4. Economic constraints during referrals	Referral often inevitable which entails additional expenses
	Few adequately staffed and equipped facilities nearby
	Limited finance to cater for accommodation/medications

#### Perception of TBAs as culturally acceptable and competent community health workers

Findings from the group discussions indicate that many families opt for traditional birth attendants as their first line of care for delivery unless they believe that labor is not normal. The fact that they are familiar and have trust in the traditional birth attendants’ ability to handle ‘normal’ deliveries was an important consideration in deciding place of delivery.

*If my wife goes into labor*, *the first thing I would do is call a traditional birth attendant*. *If she* (*traditional birth attendant*) *believes that the labor can be managed at home*, *we will stay at home*. *We will go to a health center only if the traditional birth attendant says so*. *We have confidence in them*. *Hence I comply with whatever the traditional birth attendant tells me to do to save the life of my wife*.

Male FGD participant 3

It is interesting to note that one of the reasons for preferring traditional birth attendants is the fact that mothers get the much needed support from their spouses and families’ presence in home deliveries. Besides, some traditional birth attendants attend to some of the longstanding traditional practices which are rooted in the beliefs and cultures of the society (such as massaging the abdomen with butter and burying the placenta around home). Discussions with both mothers and fathers suggest that traditional birth attendants’ approach fulfils the expectations of laboring mothers and their immediate families in a way the modern health system does not.

*According to our tradition*, *the placenta should be buried around our home*. *It* (*the placenta*) *should by no means be thrown out everywhere*. *However*, *if mothers deliver in health facilities*, *the health professionals will not give us the placenta*. *Hence*, *most of us do not prefer to go to health facilities*.

Male FGD participant 7

*Women prefer the traditional birth attendants than going to health facility because when they attend deliveries*…. *First*, *they will examine the abdomen of pregnant mothers and if they found out that the baby is not in the* ‘*right position*’, *they apply butter and do massage on the abdomen to bring the baby to its* ‘*normal position*’ *which facilitates smooth delivery*.

Female FGD participant 6

The interviews reflected that there is no clear relationship between traditional birth attendants and the formal health system. As the following quote shows, traditional birth attendants rely on the respect and trust earned from their communities through their service.

*We don*'*t have any relationship with health workers in health centers and health posts*. *They don*'*t want us*.…*And we don*'*t get any support from them*. *However*, *the community believes in us and that is our major source of support and motivation to continue the work we are doing*.

Traditional birth attendant 1

#### Perceived quality of care and previous experiences

Women who preferred to deliver at home indicated that some health professionals are not sensitive to their privacy and care little to give them psychological support when they need it most. As the following quote illustrates, families seem to value the more supportive and comfortable care that they get from traditional birth attendants.

*I am afraid of delivering in a health facility*. *They* [*health professionals*] *don*’*t allow my family members* (*who are the main source of psychological support and comfort*) *to accompany me in the labor ward*. *They leave us alone on the delivery couch and everybody who comes in and out of the delivery room watches our naked body which is quite embarrassing*. *We also get little respect from health workers*. *I won*’*t have these problems if I go to a traditional birth attendant*. *I seek help from a health facility only as a last resort* (.*i*.*e*., *if I encounter difficulty to deliver in my home*).

Female FGD participant 2

Comments from group discussion participants point to concerns about quality of care at health facilities and the lack of confidence in health workers’ ability to deal with pregnant and laboring mothers. Besides, many of the discussants mentioned that the health centers may not be open during nights and weekends.

*My last child was born at home and I didn*’*t take my wife to any health facility*. *This is because of what happened during her pregnancy check up*. *We were told that it was twin pregnancy which*, *to our surprise*, *was later confirmed to be* ‘*single*’ *in a different facility*… *Fortunately*, *she delivered at home safely and her mother attended the labor*.

Male FGD participant 4

*I had repeated antenatal visits during my first pregnancy*. *In one of my visits* (*at the ninth month of pregnancy*), *I experienced a severe crampy abdominal pain for which the health care provider advised me to take medications assuming it is caused by intestinal worms*. *Lately*, *I realized that I was actually in true labor and was thus forced to have my first child delivered at home*. *That experience eroded my confidence and trust on health professionals*’ *competence as a result of which I decided to deliver at home for all of my subsequent children*.

Female FGD participant 1

*As it is known*, *labor often comes during the night*. *People coming from rural villages pay a lot of money for transportation*. *However*, *when we reach there*, *it is possible that the facility is closed*. *Even if they are open*, *medications and equipment are often in short supply*. *Eventually*, *we end up taking prescriptions to buy from private pharmacies*. *Some health workers who are on night duty are also not competent enough to manage deliveries*. *Many times*, *they refer all laboring mothers to other hospitals*. *We face similar problems during weekends*. *These are serious problems which discourage us from going to a health facility in the first place*.

Male FGD participant 1

Although many of the study participants pointed out the advantages of traditional birth attendants, a few participants were critical of their service mainly because of safety concerns.

*I prefer to deliver my children in a health facility*. *Because the traditional birth attendants do not wear gloves and they may not as well use clean equipments*. *In contrast*, *health care providers wear gloves*, *use clean equipments and medications for delivery*.

Female FGD participant 7

*All my children were born in a health facility*. *Health facility delivery will benefit mothers as well as their children*. *In contrary*, *traditional birth attendants can sometimes be careless in managing deliveries which could endanger the health of mothers and their babies*. *That is why I preferred to deliver my last child in health facility*.

Female FGD participant 8

Interviews with health care providers indicate that some of the reasons for not coming to health facilities are results of unfounded rumors about quality of care at health facilities.

*We learned that there are rumors in the community claiming that every mother who goes to a health facility gets operated*. *Of course this kind of misperception is improving from time to time and we are getting the publics*’ *trust as witnessed by a lot of mothers coming to us*. *Many people are now convinced that we make decisions depending on the kind of problem the mothers are facing*.

Health care provider 1

#### Low awareness and less empowered women

There seems consensus among the health workers that knowledge about the importance of delivering in health facilities is fairly low. Communities often use their uneventful previous experiences to strengthen their claim that modern health care may not be necessary for delivery.

*All my children were born at home assisted by traditional birth attendants*. *In this locality*, *once a woman delivers her first child at home*, *the rest of her children will also be delivered at home*. *This is because mothers believe that once they are able to give their first birth at home nothing will happen to them in their consecutive home deliveries*. *In general*, *in this area mothers deliver their children in their home and no woman wants to deliver her child in health facility unless they face difficulty*.

Female FGD participant 2

It was suggested that many elderly women promote traditional health care at times advising against services at health facilities. As opinion leaders especially on matters related to labor, their position influences the care seeking behavior of particularly young mothers.

*We still have many elderly people who discourage going to a health facility for labor claiming that they used to have a better past when there were no modern healthcare services including vaccination*, *and family planning*. *For them the health facilities have little impact*, *if any*. *These influential people need to be engaged if we are to improve the health seeking behavior of others*.

Volunteer Community health worker 6

*In most situations*, *it is relatives and neighbors* (*including traditional birth attendants*) *who are the main decision makers in this community*. *And this is one of the reasons why laboring mothers stay at home during delivery*.

The discussions also point that the community’s awareness is improving from time to time. As the following participant explains more and more women are going to health facilities with increasing awareness and better access to health posts in their own villages.

*Women in our area usually deliver at their home*. *One of the reasons is the fact that there were very few health facilities in our locality which provide delivery service*. *However*, *currently health posts are being constructed in many villages* (‘*kebeles*’) *and there is also ongoing education by different health workers about the importance of delivery at health institutions which improved service utilization in recent times*.

Male FGD participant 1

#### Economic constraints during referrals

Cost is a significant deterrent of maternity service seeking in many rural settings. Where distance to the next level of care is a barrier, indirect cost to cover transportation and lodging expenses become particularly important. As illustrated by the following quotes, one of the reasons for not going to the closest health centers appears to be the anticipation of inadequately equipped facilities and further referrals to more distant hospitals which is hardly affordable.

*If a laboring mother is referred to other hospitals*, *she needs to have at least 800 Ethiopian birr for transportation*. *Most people cannot afford this amount of money*. *It is usually during this time that the fetus dies due to* ‘*distress*’ *and the mother also gets extremely weak*. *Health facilities are not well equipped with facilities and there is also shortage of essential drugs*.

Male FGD participant 1

*One of the major factors which hinder women from going to health facilities is their financial problem*. *Even though they know the importance of institutional delivery*, *they may not go to health facilities as they know they will be referred*. *Most of the time*, *they borrow money from their relatives*. *However*, *getting that money takes days and it*’*s during this time that the labor goes into complications*.

Male FGD participant 2

*Even when families know that the labor is not progressing well and the laboring mother is not doing well*, *they stay up to 3 days until they exhaust all possibilities* (*before they sell assets to cover transportation and accommodation expenses*). *Such financial constraints often prevent mothers from seeking timely care*.

Community health volunteer 1

## Discussion

The present study examined factors affecting women’s preference to home delivery in a predominantly rural region in Ethiopia where physical and financial access to a health facility have been addressed to a certain extent. The study gives in-depth insight into a range of factors that hinder facility based delivery which are often described as lack of knowledge and misperceptions in quantitative surveys. As expected women who were educated and those who had better autonomy in decisions pertaining to household expenses were more likely to deliver in health centers. The findings of the survey and qualitative interviews and discussions are largely similar in that financial and transportation problems are mentioned as less important reasons to access care from the nearby health centers which is unlike other studies [11,12].

Our study shows that service availability and physical access do not always result in good delivery service utilization which is in agreement with previous reports [22,23]. Globally, home birth is increasing even in high income countries where service affordability is not a big factor in getting institutional delivery [24]. Reasons for these emerging trends include the fact that issues that enhance the experience of mothers including convenience, privacy, and respect and providing room for immediate family members (partner, or mother or other designated person) are addressed in a better way by home deliveries [25-31].

The findings indicate that to some extent women’s preference for home delivery has been shaped by their negative past experiences with the modern health system including health care providers’ competence and poor availability of drugs and equipments [26]. It was also noted that some women do not go to health facilities anticipating that the facilities will not be fully functional outside the normal working hours (at nights and weekends).

The study results highlight the importance of responsiveness of care in affecting delivery service utilization even in predominantly rural and low resource settings. Various studies have reported barriers embedded in the beliefs, and traditions of communities including poor family participation, fear or embarrassment related to receiving care at health facilities, as well as the perception that health professionals are not paying sufficient attention to traditional norms of the society [31-37].

Equally important is the relatively strong position of traditional birth attendants as culturally acceptable and trusted source of service providers, who apparently do not have formal linkages with the modern health care and no incentives for early referral and communication. This is in accordance with other studies which reported traditional birth attendants as preferred service providers in rural areas because of their cultural sensitivity, easy availability and cheaper services [38-40].

While the Ethiopian health system acknowledges the role of traditional birth attendants as volunteers working under the supervision of the health extension workers [41], their relationship has not been clearly defined. Experiences from other countries indicate that skilled attendance can be better promoted in a system that integrates traditional with the modern health system [35]. Although the evidence is mixed [42], evaluations of programs which promote trained traditional birth attendants have indicated that their service could increase women’s use of antenatal care and emergency obstetric care, and decrease perinatal and neonatal mortality in a context where access to health services is limited and maternal mortality is high [43].

One of the strengths of the present study is that it employed both questionnaire survey and qualitative approaches among a range of stakeholders; women, fathers, community health workers, traditional birth attendants and health care providers which allowed triangulation of findings from multiple perspectives. A potential limitation could be that the study was conducted in only three districts in the Southern region which are within 200 km radius from the national capital and thus may not represent what could be prevalent in more remote corners of the country. It is possible that some of the cultural barriers for service utilization can be context specific limiting transferability of the findings.

## Conclusions

### Implications for development of a guideline for managing laboring mothers in health facilities and promoting patient centered care

The study indicated that there is a need to emphasize the importance of health care provider-client communication and client centered care in both pre-service and in-service trainings so that women get the respectful attitude and supportive environment that they usually claim to get from traditional birth attendants.

The way expectant mothers and their immediate families are treated varies a lot from facility to facility and there are no guidelines regarding issues such as privacy and psychological support which could be key in determining service utilization. A strategy towards addressing this challenge could be to design and implement a standard protocol across all health facilities regarding the handling of mothers and their relatives during labor. This will help improve mothers’ experience during labor without affecting the quality of care.

The fact that there is no proper communication between the different levels of care has also meant that laboring mothers in need of immediate medical attention may avoid going to health facilities anticipating that the facilities will be closed or not functional particularly outside of the conventional working hours. While it is important to avail emergency obstetric services in the nearest health facilities, the communication between the health facilities should be improved to avoid unnecessary referrals and alert the next level health care providers about incoming patients.

### Implications for involving TBAs and designing more context specific educational messages for the community

Given the fact that traditional birth attendants are still regarded well by their communities attending the majority of births, it is important to engage them with the formal health system and facilitate their role in early referral and possibly dispelling misperceptions surrounding labor and delivery in their localities. On the basis of the current and previous studies, it is imperative that future programs consider innovative incentive mechanisms (which could be non-monetary; for example in the form of recognition) for traditional birth attendants who are active in referring mothers to health facilities.

The study pointed that some reasons for not seeking delivery care (such as the traditional values attached to bury the placenta closer to their homes) could be highly context specific which means information education and communication interventions targeting behaviour change should address such socio-cultural beliefs of communities.

### Implications for further research

Finally, the study revealed that some of the important reasons for preferring home over facility based delivery cannot be captured in the usual structured questionnaires of community based surveys. These factors are commonly categorized as ‘unnecessary’ and ‘not customary’ in large quantitative assessments including the demographic and health surveys indicating the importance of using qualitative approaches to get a more complete picture and design more specific educational messages. Future studies should also explore for important motivation factors that can help improve communication between traditional birth attendants and the formal health system.

## Competing interests

The authors declare that they have no competing interests.

## Authors’ contributions

SS designed the study, coordinated the data collection and management, and helped to draft the manuscript. YM participated in design and analysis of the study. MT participated in coordinating the field work and drafting the report. MS involved in drafting and revising the manuscript. MB participated in qualitative data analysis and revising the manuscript. All authors read and approved the final manuscript.

## Pre-publication history

The pre-publication history for this paper can be accessed here:

http://www.biomedcentral.com/1471-2393/13/5/prepub
